# The Spatial Origin of a Perceptual Transition in Binocular Rivalry

**DOI:** 10.1371/journal.pone.0002311

**Published:** 2008-06-11

**Authors:** Chris L. E. Paffen, Marnix Naber, Frans A. J. Verstraten

**Affiliations:** Helmholtz Institute and Experimental Psychology, Utrecht University, Utrecht, The Netherlands; University of Southern California, United States of America

## Abstract

When the left and the right eye are simultaneously presented with incompatible images at overlapping retinal locations, an observer typically reports perceiving only one of the two images at a time. This phenomenon is called binocular rivalry. Perception during binocular rivalry is not stable; one of the images is perceptually dominant for a certain duration (typically in the order of a few seconds) after which perception switches towards the other image. This alternation between perceptual dominance and suppression will continue for as long the images are presented. A characteristic of binocular rivalry is that a perceptual transition from one image to the other generally occurs in a gradual manner: the image that was temporarily suppressed will regain perceptual dominance at isolated locations within the perceived image, after which its visibility spreads throughout the whole image. These gradual transitions from perceptual suppression to perceptual dominance have been labeled as traveling waves of perceptual dominance. In this study we investigate whether stimulus parameters affect the location at which a traveling wave starts. We varied the contrast, spatial frequency or motion speed in one of the rivaling images, while keeping the same parameter constant in the other image. We used a flash-suppression paradigm to force one of the rival images into perceptual suppression. Observers waited until the suppressed image became perceptually dominant again, and indicated the position at which this breakthrough from suppression occurred. Our results show that the starting point of a traveling wave during binocular rivalry is highly dependent on local stimulus parameters. More specifically, a traveling wave most likely started at the location where the contrast of the suppressed image was higher than that of the dominant one, the spatial frequency of the suppressed image was lower than that of the dominant one, and the motion speed of the suppressed image was higher than that of the dominant one. We suggest that a breakthrough from suppression to dominance occurs at the location where salience (the degree to which a stimulus element stands out relative to neighboring elements) of the suppressed image is higher than that of the dominant one. Our results further show that stimulus parameters affecting the temporal dynamics during continuous viewing of rival images described in other studies, also affect the spatial origin of traveling waves during binocular rivalry.

## Introduction

When the two eyes are confronted with dissimilar images, each of the two images undergoes alternating periods of perceptual dominance and suppression. This phenomenon is called binocular rivalry and is highly popular among vision scientists since it has been argued that it can provide insights into the neural correlate of consciousness [1]. In addition, it has proven to be a useful tool to study various aspects of visual processing [2] and efforts are made to understand what processes give rise to the phenomenon [3]. For example, perceptual alternations during binocular rivalry generally occur automatically and are subject to limited voluntary control [4]–[6]. This finding is in correspondence with the hypothesis that rivalry is instigated at early levels of visual processing [7], although this hypothesis is under debate [3].

A characteristic of binocular rivalry is that a rival image will completely dominate perception only under a limited set of conditions, a situation referred to as exclusive visibility. The size of both images for example, affects the amount of exclusive visibility: Blake, O'Shea and Mueller [8] estimated that rival images both having diameters up to 8.1 min of arc lead to exclusive visibility about 95% of the time. With increasing size of the images, the incidence of exclusive visibility decreased. Furthermore, the maximum size of rival images leading to exclusive visibility increases with retinal eccentricity [8] and decreases with increasing spatial frequency [9], [10]. Also, the incidence of exclusive visibility will decrease with prolonged viewing time [11] and contrast [10], but will increase with shared stimulus complexity [12]. As dominant perception of one of the rival images is seldom exclusive, the same holds for a transition from one dominant image to the other; a perceptual transition during rivalry generally does not occur in an all-or-nothing fashion. Specifically, it has recently been appreciated that the transition from one dominant percept to the other can occur in a wave-like fashion [13]. These traveling (or dominance) waves propel at a fixed speed when corrected for cortical magnification at different visual eccentricities [13]. Interestingly, the speed of the traveling waves correlates with neural propagation speed in V1, V2 and V3 [14], [15].

**Figure 1:**
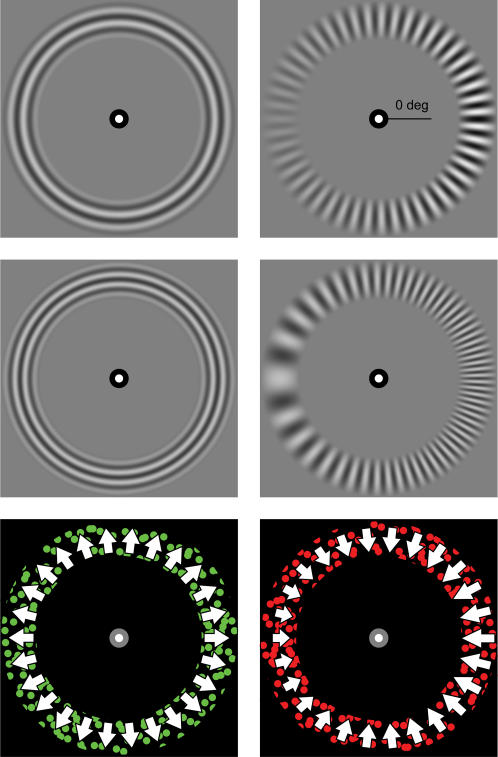
Stimuli used in the three experiments. The top row depicts the stimulus used in Experiment 1 (contrast), the middle row the stimulus in Experiment 2 (spatial frequency) and the bottom row the stimulus in Experiment 3 (motion speed). For each row, the left image was presented to one eye, the right image to the other (the presentation was counterbalanced). In each of the experiments, the parameter under study was constant in one image (the CONST image; left column), while the parameter was varied in the other image (the VAR image; right column). The upper right image indicates the 0 deg position.

**Figure 2:**
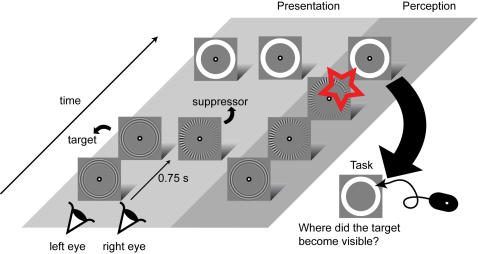
General procedure. In short, one image was flash-suppressed (the target, shown in the most left stream of events) by another image (the suppressor, shown in the middle stream of events). When the suppressor appeared, the target became invisible (most right stream of events). An observer then waited until the target became visible again upon which he or she was instructed to click a mouse button at the location where the target reentered perception.

In this study we ask whether the spatial origin of a transition from perceptual suppression to dominance, and thus the starting point of a traveling wave, is influenced by contrast, spatial frequency and motion speed. To examine these stimulus parameters, we placed rival images at overlapping retinal locations using a mirror stereoscope. In three experiments, contrast, spatial frequency or motion speed, was varied in one of the rival images (the VAR image), while the parameter was fixed in the other image (the CONST image; see Figure 1). In order to investigate whether the spatial origin of a transition from suppression to dominance was influenced by these parameters, we investigated two basic conditions in each of the experiments. In one condition, observers indicated where a transition from a CONST to a VAR image (CONST-to-VAR) started. In the other condition, observers indicated where a transition from a VAR to a CONST image (VAR-to-CONST) started. In order to investigate these conditions, we had to make sure that the VAR image would be suppressed in the CONST-to-VAR condition, and that the CONST image would be suppressed in the VAR-to-CONST condition. We used the flash-suppression paradigm ([16] see Figure 2) to achieve this goal.

Flash-suppression was achieved in the following manner. First, a single image (hereafter named the target) was presented to a single eye. A few moments later (0.75 s) the rival image (hereafter named the suppressor) was presented to the corresponding retinal location of the other eye, while the target remained present. When an observer viewed these events, the target would be perceived up to the moment that the suppressor was presented. At that moment, the percept would switch from the target to the suppressor, which we designate as “target suppression”. A short time later the target would become visible again, since conventional binocular rivalry would start from the moment of target suppression. At that point, the observer indicated the position where the target became dominant again, after which a trial ended.

During continuous viewing of rival images, stimulus parameters affect the average dominance duration of each of the images. For example, when binocular rivalry is instigated between a low and a high contrast grating, the high contrast grating usually has a longer dominance duration than the low contrast one [17]–[19]. For spatial frequency, there is no clear relationship between a given spatial frequency and the average dominance duration [20], although it has been reported that the average dominance duration is lower for rival images with isolated spatial frequencies than for unfiltered broadband images [20], [21]. Motion has also been found to influence the average dominance duration during rivalry: images containing motion have longer dominance durations than static ones [22], [23] and higher speeds have higher dominance durations than lower ones [23]. As a theoretical construct, stimulus parameters that increase dominance durations in rivalry are said to have higher stimulus strength [24]. We hypothesized that parameters that affect dominance durations during continuous viewing of rival images (e.g. higher contrast or higher motion speed) also affect the spatial origin of a perceptual transition from one image to the other. Thus, we expect that a perceptual transition from one rival image to the other one will start at the location where the stimulus strength of the suppressed image is higher than that of the dominant one.

## Materials and Methods

### Observers

A total of five observers, four naïve and one author, participated in the experiments and all observers had normal, or corrected to normal visual acuity. The experiments were carried out with the understanding and written consent of each observer. The experiments were approved by the Medical Ethical Committee of the Faculty of Social Sciences of Utrecht University.

### Stimuli & Apparatus

The stimuli were created and presented using MATLAB in conjunction with the PsychToolbox, using an Apple PowerMac G4 (Experiment 1 & 2) and an Apple PowerMac G5 (Experiment 3) on a linearized LaCie III 22' at 85 Hz (Experiments 1 & 2) and 120 Hz (Experiment 3). The stimuli were presented within an annular aperture with an inner radius of 2 deg and an outer radius of 3 degs (degrees of visual angle; Figure 1). The edges of the stimuli in Experiments 1 & 2 were filtered by half a period of a raised cosine with a width of .5 deg. The parameter under study (e.g. contrast) in the VAR image increased as a biphasic linear function of angle along two halves of the annulus, where the variable reached its maximum 180 degrees from its minimum. Importantly, the maximum value of the parameter in the VAR image was presented at 0, 90, 180 or 270 degrees clockwise from right horizontal (see Figure 1). This strategy was adopted to exclude the possibility that systematic biases for spatial positions were due to spatial locations per se, and not to variations in stimulus parameters. The background luminance of the monitor was 25.2 cd m-2 for Experiments 1 & 2, and <0.1 cd m-2 for Experiment 3.

#### Stimuli: Experiment 1 (Figure 1 top row)

The rival images in the contrast experiment were sine-wave gratings, where the CONST image consisted of a concentric grating with a fixed contrast of 59.4% (Michelson). The VAR image consisted of a radial grating with a contrast varying between 12% and 100% (Michelson). The spatial frequency of both images was fixed at 3 cpd.

#### Stimuli: Experiment 2 (Figure 1 middle row)

In the spatial frequency experiment, the CONST image was a concentric grating with a fixed spatial frequency of 3.9 cpd. The VAR image was a radial grating with a spatial frequency varying between 0.9 and 6.9 cpd. Michelson contrast of each of the images was fixed at 59.4%.

#### Stimuli: Experiment 3 (Figure 1 bottom row)

The CONST motion image consisted of dots moving inward or outward at a fixed speed of 3.3 deg s-1. The VAR motion image consisted of dots moving inward or outward at a speed that varied between 0.5 and 6.0 deg s-1. The speed of the individual dots was varied by varying the step size of the individual dots. Dots in both images had a lifetime of 40 frames. On average, each image contained 400 dots per frame. At each trial, dots in one of the images were green and dots in the other red. Also, one of the images contained inward and the other one outward motion (presentation of these features was counterbalanced). The green and red dots were presented at the observers' perceptual isoluminance, which was acquired by using a flicker-matching procedure (by matching red to green (green: luminance 14.6 cd m-2, x = .292, y = .607)).

### Procedure

The general procedure is schematically illustrated in Figure 2. An observer initiated a trial by pressing the space bar. Next, the target image was presented to either the left or the right eye; 750 ms later, the suppressor was presented to the other eye, resulting in perceptual dominance of the latter image. Subsequently, the observer waited until the target became visible again. As soon as this happened, the observer moved the computer mouse to the position where the target regained perceptual dominance and clicked at this position. The two basic conditions, CONST-to-VAR and VAR-to-CONST were randomly interleaved. The eye to which the target was presented was counterbalanced. In Experiment 3, the type of motion (inward or outward) as well as the color of the target image were also counterbalanced. In total, each subject performed 80 trials per condition (for CONST-to-VAR as well as for VAR-to-CONST conditions) for the contrast and spatial frequency experiments, and 96 trials per condition for the motion experiment.

## Results

**Figure 3:**
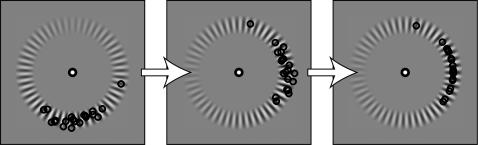
Transformation of the data. Data for each condition were rotated to represent the data as corresponding to the highest value of the stimulus parameter at the 0 deg position. Next, data were put on a unit circle and convoluted with a Gauss (see Result for details).

**Figure 4:**
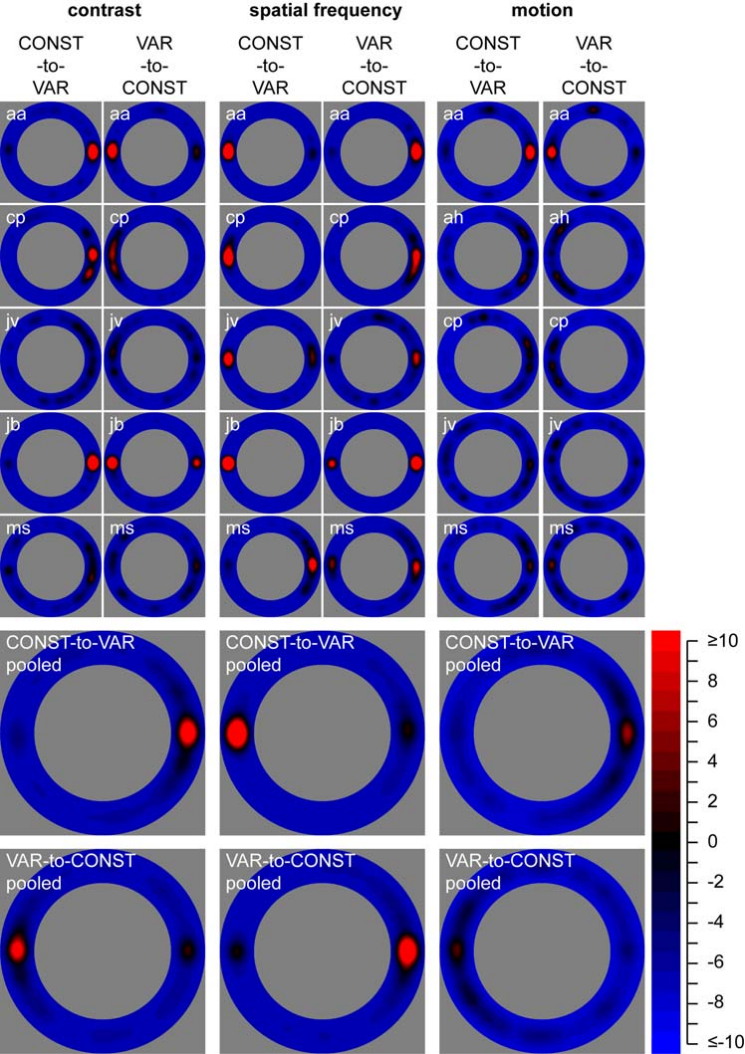
Results. The first five rows show results of individual observers for each of the experiments. For each experiment, the left column represents z-scores for the CONST-to-VAR conditions, and the right column represents z-scores for the VAR-to-CONST conditions. The bottom two rows show pooled results for each of the experiments. Here, the first row of data represents z-scores for the CONST-to-VAR conditions for each of the experiments; the second row of data represents z-scores for the VAR-to-CONST conditions for each of the experiments. Importantly, the data represent most frequent mouse clicks for data rotated to the 0 deg position (as explained in the Results section). The colors in the graphs represent z-scores, where red indicates positive and blue negative z-scores.

Before analyzing the results, data of the four stimulus configurations (where the maximum of the stimulus parameter in the VAR image was presented at either 0, 90, 180 or 270 degrees clockwise from right horizontal) were rotated back to the 0 deg positions (from Figure 3, left square to Figure 3, middle square). Next, these data were re-positioned on a unit circle to represent the data on an annulus with fixed radius (Figure 3, right square). This transformation was performed for all stimulus configurations for all experiments. Next, the transformed data were convoluted with a Gaussian with amplitude of 1 and a σ of 0.2 deg. Peaks in this distribution now indicate zones of the most frequent mouse clicks. To find out how these distributions differed from chance, we performed a simulation where points (80 for the contrast and spatial frequency, and 96 for the motion speed simulation) on the circle were randomly drawn and where also convoluted with a Gaussian (amplitude of 1, σ of 0.2). This simulation was run 1000 times, from each taking the value of the highest peak. The mean and standard deviation of these peaks were used to calculate z-scores that are illustrated in Figure 4.

We report here on the mean z-scores of five observers (last two rows of Figure 4). For the CONST-to-VAR conditions, a perceptual transition most often started at the location where the contrast of the VAR image was highest (z = 22.5), the spatial frequency of the VAR image was lowest (z = 35.7) and the motion speed of the VAR image was highest (z = 6.9). For the VAR-to-CONST conditions, a perceptual transition most often started at the location where the contrast of the VAR image was lowest (z = 15.4), the spatial frequency of the VAR image was highest (z = 26.9), and where the motion speed of the VAR image was lowest (z = 3.9). For the stimulus defined by a contrast gradient in the VAR-to-CONST condition, a perceptual transition also started significantly above chance at the location where the contrast of the VAR image was highest (z = 2.6). For all other locations in the two conditions of the three experiments, the probability of reporting the start of a perceptual transition was less than two standard deviations from the simulated average (or z<2, see Appendix 1). This indicates that density of mouse clicks due to spatial biases unrelated to stimulus parameters did not differ significantly from chance in our experiments.

## Discussion

Our experiments show that the spatial origin of a perceptual transition is highly dependent on stimulus parameters. In each of the experiments, the contrast, spatial frequency or motion speed influenced where in the perceived image a transition started. Importantly, the starting point of transition was influenced by stimulus parameters in the CONST-to-VAR conditions, as well as in the VAR-to-CONST conditions (see Figure 4, last two rows). Thus, the spatial origin of a transition was influenced both when the VAR image was the target (when a transition from the CONST to the VAR image was monitored) and when it was the suppressor (when a transition from the VAR to CONST image was monitored). This result indicates that it is the difference in the stimulus attribute under study that determines where a target will break suppression. For contrast and motion speed, a perceptual transition most often occurred at the location where the value of the parameter of the target (contrast or motion speed) was higher than that of the suppressor. For spatial frequency, this location was where the spatial frequency of the target was lower than that of the suppressor. These results have implications for models on binocular rivalry [7], [25]–[28], which should incorporate the finding that a perceptual transition can occur at specific locations within the rival image. More specifically, most models contain monocular representations of the rival images. These representations should allow for variable levels of reciprocal inhibition related to local stimulus characteristics.

In most studies on binocular rivalry, observers continuously report on their dominant percept of two rival images. The concept of stimulus strength, introduced by Levelt [24], is often used in these studies to assess the perceptual strength of rival images. In Levelt's terms, an image with a longer dominance duration than its rival has higher *strength*. For example, a high contrast image has high strength, since its dominance duration is usually longer than that of a low contrast rival image [17]–[19], and a high motion speed image has high strength since its dominance duration is longer than that of a low motion speed rival image [23]. Do stimulus parameters with high strength dictate at what location a perceptual transition starts in binocular rivalry? For contrast and motion speed, the answer appears to be positive: our results show that a transition most often occurred at the location where contrast or motion speed of the target was higher than that of the suppressor. For spatial frequency, the answer is unclear since there is no clear relationship between isolated spatial frequencies and average dominance duration [20], and thus between spatial frequency and strength. At present, we have no explanation for the fact that varying spatial frequency does modulate the spatial origin of a perceptual transition (our results), but does not modulate average dominance duration during continuous viewing of rival images.

What could be a general rule determining where a perceptual transition originates in binocular rivalry? The concept of stimulus strength only seems applicable to our results on contrast and motion speed and not on spatial frequency. An obvious hypothesis is that a transition during binocular rivalry starts at the location where sensitivity to the parameter under study is highest. For spatial frequency, sensitivity is often assessed by measuring contrast discrimination or detection thresholds for stimuli of different spatial frequencies and usually peaks around 2–4 cpd [29]. For motion speed, sensitivity has been assessed by measuring the strength of the motion aftereffect, and peaks between 1 and 6 deg/s [30]. Clearly, locations at which sensitivity should be highest (around spatial frequencies of 2–4 cpd and around motion speeds of 3.5 deg/s) were not the locations where transitions started most often. We propose that saliency is a better candidate in predicting the spatial origin of a perceptual transition. The concept of saliency is often used in attention research to describe the degree to which an element stands out relative to its neighboring elements (the reader can appreciate that the locations with the highest contrast and the lowest spatial frequency are the most salient locations in Figure 1). Note that the concepts stimulus strength, sensitivity and saliency appear to be similar, although they are not. The concept of stimulus strength is restricted to studies on binocular rivalry and is used to describe the relative dominance of one rival image over the other. Sensitivity is used to assess the degree to which the (visual) system is sensitive to a stimulus parameter under study. Saliency refers to the degree to which a stimulus element stands out relative to its neighboring elements. For example, if a letter T is surrounded by multiple letters L, the T is a salient element in this stimulus, although sensitivity to the two letters would presumably not be different. It has been suggested that saliency of a visual scene is computed pre-attentively in primary visual cortex [31]. For example, firing rates of V1 neurons increase monotonically with the saliency of the visual input [32], [33]. At the same time, many studies show a crucial role of V1 in binocular rivalry [14], [34], although higher-level processing areas have also been implicated [35]–[37]. From this, it is to be expected that the dynamics of binocular rivalry are subject to manipulations in saliency of the rival images. Indeed, Bonneh and Sagi [38] showed that, in short duration binocular rivalry, configuration saliency affects perceptual dominance. Based on our results, we suggest that saliency can be computed on a monocular level (a suggestion also made by [38]) without awareness, since the saliency of a monocular - suppressed - image determined where the image regained perceptual dominance. This suggestion is line with views proposing that saliency is computed preattentively, at early levels of visual processing [31], [39], [40].
